# The rate of acute kidney injury (AKI) alert detection by the attending physicians was associated with the prognosis of patients with AKI

**DOI:** 10.3389/fpubh.2022.1031529

**Published:** 2022-11-17

**Authors:** Yu Shi, Hai Wang, Ling Bai, Yuan Wu, Li Zhang, Xin Zheng, Jun-hua Lv, Hong-hong Pei, Zheng-hai Bai

**Affiliations:** ^1^Emergency Department, The Second Affiliated Hospital of Xi'an Jiaotong University, Xi'an, China; ^2^Department of Hepatobiliary Surgery, The First Affiliated Hospital of Xi'an Jiaotong University, Xi'an, China

**Keywords:** acute kidney injury, electronic alert, AKI alert, attending physicians, prognosis

## Abstract

**Introduction:**

Early identification of AKI was always considered to improve patients' prognosis. Some studies found that AKI early warning tools didn't affect patients' prognosis. Therefore, additional studies were necessary to explore the reasons.

**Methods:**

This study was a secondary analysis of a multicenter randomized controlled trial that found electronic health record warnings for AKI did not influence patients' prognoses. Univariate, multivariate, subgroup, curve fitting, and threshold effect analysis were used to explore the association between AKI warnings detected by attending physicians and the patient's prognosis.

**Results:**

A total of 6,030 AKI patients were included in the study. The patients were classified into two groups based on the rate of AKI alerts detected by attending physicians: the partial group (*n* = 5,377), and the complete group (*n* = 653). In comparison to the partial group, the complete group significantly decreased 14-day AKI progression, 14-day dialysis, and 14-day mortality, with adjusted ORs of 0.48 (0.33, 0.70), 0.26 (0.09, 0.77), and 0.53 (0.33, 0.84) respectively, and the complete group significantly improve the discharge to home, with an OR value of 1.50 (1.21, 1.87). When the rate of AKI alerts detected by the attending physicians as a continuity variable, we found that the rate of alerts seen by attending physicians was associated with 14-day mortality and the discharge to home, with adjusted ORs of 1.76 (1.11, 2.81) and 1.42 (1.13, 1.80). The sensitivity analysis, curve-fitting analysis, and threshold effect analysis also showed that the rate of alert seen by the attending physician was correlated with the patient's prognosis.

**Conclusion:**

The rate of AKI alert detection by attending physician were related to the patient's prognosis. The higher the rate of AKI alert detection by attending physicians, the better the prognosis of patients with AKI.

## Introduction

AKI is a prevalent clinical syndrome with a significant incidence and significant mortality risk (1–4). Patients' outcomes might be vastly improved with early detection and treatment of AKI, which had been widely believed for a very long time (5–7). The clinical practice used to often be accompanied by a delayed diagnosis of AKI since there were no techniques available to warn of AKI from time to time (8). According to reports, more than 25% of hospitalized patients with a doubled creatinine level documented AKI without a symptom record, and unrecorded AKI was independently associated with a higher mortality rate (9). Excitedly, automated early warnings based on electronic medical data may effectively counter the false reports or alarms of AKI (10, 11). Unfortunately, related research showed that the automatic early warning model of AKI did not seem to improve the prognosis of patients. A study of 1,201 individuals with AKI randomly assigned them to the early warning group or the usual treatment group in a ratio of 1:1. The research discovered that an electronic alarm system for acute kidney damage did not improve hospital patients' clinical outcomes (12). A randomized controlled trial with 6,030 AKI patients, similarly demonstrated that the automated early warning model based on electronic medical data could not enhance the prognosis of AKI patients (13). Early detection of AKI might improve the patient's prognosis by changing the dosage of medication properties, avoiding nephrotoxicity, and paying attention to fluid balance, which needed the attending physician to develop a systematic and comprehensive treatment strategy for the patient (14). Therefore, the impact of AKI early warning on the patient's prognosis might be related to the rate of AKI early warning identification by the attending physicians. However, there is no relevant research to explore the relationship between the rate of AKI alert detection by attending physicians and the patient's prognosis. Therefore, this study assumed that the identification rate of AKI early alerts by the attending physician might be one of the main reasons, which influenced the effect of early warning of AKI on the prognosis of patients.

### Objective

To investigate the association between the rate of AKI alerts detected by the attending physicians and the prognosis of patients with AKI including 14-day AKI progression, 14-day dialysis, 14-day mortality, and the discharge to home.

## Methods

### Study design

An exploratory study of a multicenter, randomized clinical trial on AKI early warning system.

### Data source

The data was taken from the digital repository at dryad. The database is a public repository of data that authors had added to, so that their research data can be found, used for free, and cited. This URL may be used to obtain further information: https://datadryad.org/stash/dataset/doi:10.5061/dryad.4f4qrfj95 (13).

### Setting

Six hospitals in the Yale New Health System in Connecticut and Rhode Island, US.

### Inclusion criteria

(1) The age of the inpatient was equal to or more than 18 years old. (2) Inpatient diagnosed with AKI according to the KDIGO (Kidney Disease: Improving Global Outcomes) AKI criteria; (3) For patients admitted for multiple times, only the data of the first admission were included in the analysis.

### Exclusion criteria

(1) patients who had previously been on dialysis; (2) patients who have end-stage renal disease; (3) patients who had an initial serum creatinine level of < 4.0 mg/L; (4) patients who are currently receiving hospice care; and (5) patients who are scheduled to undergo kidney transplantation within the next 6 months.

### The definition of AKI in electronic early warning system

A creatinine rise of 0.3 mg/dL (26.5 mol/L) within 48 h, or 1.5 times the lowest measured creatinine during the preceding seven days of hospitalization.

### The electronic alert system for AKI

The AKI diagnostic algorithm (KDIGO AKI criteria) was built into the medical system, along with automated collection of key indications and generation of alerts. When medical staff open the medical system, an AKI “pop-up” warning displays and the indications for AKI diagnosis will also be shown to let the them to check the accuracy of the warning. If different medical staff access the same patient's medical system, they will be warned individually (13). Before the study, all medical staff obtained AKI and alert system education to guarantee appropriate and reliable application. Interns, residents, fellows, attending physicians, nurse practitioners, and physician's assistants, together referred to as “providers,” were the only ones to see alerts. Alerts were shown when the chart was opened as long as the patient still met the criteria for AKI. Whether the provider agreed or disagreed that AKI was present, the alert was turned off for that provider for 48 h. If more than one provider used the same patient's electronic health record, the alert would show up for all of them (13).

### The AKI alert detected by attending physicians

An attending physician detected an AKI warning, indicating that the AKI alert was recognized independently of individual doctors or the treatment team.

### Grouping

The patients were classified into two groups based on the rate of AKI alerts detected by attending physicians: the partial group (*n* = 5,377) in which only partial AKI alert was detected by attending physicians, and the complete group (*n* = 653) in which 100% of AKI alert was detected by attending physicians. The partial group was used as an control to calculate the risk ratios.

### The outcome indicators

14-day AKI progression (defined as an increase in AKI stage), 14-day dialysis, 14-day mortality and discharge to home.

### Statistical analysis

The mean and standard ± deviation were used to describe data for continuous variables, whereas numbers and percentages were used to describe data for counting variables. Two groups were compared using the one-way analysis of variance (ANOVA) or the Kruskal-Wallis test (K-W test) based on the continuous variables' distributions and variances. We employed the chi-square test since it was appropriate for counting variables. The association between the rate of AKI alerts detected by attending physicians and the progression and prognosis of patients with AKI was identified using univariate analysis, multi-factor regression analysis, smooth curve fitting, and threshold effect analysis. EmpowerStats (http://www.empowerstats.com, X&Y Solutions, Inc, Boston, MA) and the R (http://www.R-project.org, The R Foundation) statistical software packages were used for the analysis. We judged a statistical difference between groups at *P* < 0.05 to be significant.

## Results

### Baseline characteristics of included patients

The clinical characteristics and laboratory findings of all patients were shown in Table 1. A total of 6,030 AKI patients were included in the study. The mean age of the partial group, and the complete group were 66.69 ± 15.35 years and 69.73 ± 15.32 years, respectively. The ratio of men to women, respectively was 2,818/2,559 and 330/323 in the two groups. The complete group was with less 14-day AKI progression, 14-day dialysis and 14-day mortality, and higher discharge to home than the complete group (Table 1).

**Table 1:** The clinical characteristic of patients.

**Variables**	**Partial group (*n* = 5,377)**	**Complete group (*n* = 653)**	***P*-value**
Age	66.69 ± 15.35	69.73 ± 15.32	< 0.001
Sex (M/F)	2,818/2,559	330/323	0.366
MAP	84.72 ± 14.68	85.63 ± 14.33	0.107
**Race**			< 0.001
African American	891 (16.57%)	55 (8.42%)	
Hispanic	575 (10.69%)	45 (6.89%)	
Other	3,911 (72.74%)	553 (84.69%)	
Diabetes	2,213 (41.16%)	271 (41.50%)	0.866
Malignancy	864 (16.07%)	67 (10.26%)	< 0.001
Liver disease	789 (14.67%)	66 (10.11%)	< 0.002
Congestive heart failure	2,342 (43.56%)	316 (48.39%)	0.019
CKD	2,007 (37.33%)	283 (43.34%)	0.003
COPD	1,790 (33.29%)	274 (41.96%)	< 0.001
Alert	2,734 (50.85%)	325 (49.77%)	0.604
Bicarbonate	23.50 ± 5.18	25.58 ± 5.31	< 0.001
BUN	31.88 ± 19.11	33.46 ± 18.01	< 0.001
HB	10.68 ± 2.34	11.27 ± 2.21	< 0.001
Anion gap	12.69 ± 4.30	9.95 ± 3.85	< 0.001
K^+^	4.25 ± 0.64	4.18 ± 0.66	0.005
Na+	138.12 ± 5.29	138.45 ± 5.09	0.012
eGFR	61.59 ± 31.76	54.93 ± 29.39	< 0.001
Elixhauser comorbidity score	6.34 ± 2.87	6.13 ± 2.62	0.216
SOFA score	2.50 ± 2.13	2.06 ± 1.82	< 0.001
Any diuretic treatment	1,496 (27.82%)	178 (27.26%)	0.761
Nephrology consult	1,317 (24.49%)	120 (18.38%)	< 0.001
Aminoglycoside treatment	38 (0.71%)	2 (0.31%)	0.234
ACEI/ARB treatment	923 (17.17%)	170 (26.03%)	< 0.001
NSAIDs treatment	547 (10.17%)	53 (8.12%)	< 0.001
Contrast examination	205 (3.81%)	12 (1.84%)	0.011
Duration of alert	3,790 (70.49%)	557 (85.30%)	< 0.001
≤ 48 h	3,790 (70.49%)	557 (85.30%)	
48 h ~7 days	1,334 (24.81%)	88 (13.48%)	
>7 days	253 (4.71%)	8 (1.23%)	
14-day AKI progression	905 (16.83%)	43 (6.58%)	< 0.001
14-day dialysis	195 (3.63%)	4 (0.61%)	< 0.001
14-day mortality	491 (9.13%)	46 (7.04%)	0.077
Discharge to home	2,650 (49.28%)	347 (53.14%)	0.063

CKD, chronic kidney disease; COPD, chronic obstructive pulmonary disease; MAP, mean arterial pressure; SOFA, sequential organ failure assessment; NSAIDs, non-steroidal anti-inflammatory drugs.

### The results of univariate analysis and multi-factor regression analysis

Univariate analysis revealed that the complete group was associated with lower 14-day AKI progression and 14-day dialysis, with the OR values of 0.35 (95% CI: 0.25 to 0.48, *P* < 0.001) and 0.16 (95% CI: 0.06 to 0.44, *P* < 0.001), and that it was not associated with 14-day mortality and discharge to home, the OR values were separately 0.75 (95% CI: 0.55 to 1.03, *P* = 0.078) and 1.17 (95% CI: 0.99 to 1.37, *P* = 0.063). In the multivariate logistic regression analysis, the following variables were adjusted: age, sex, race, Na^+^, K^+^, anion gap, HB, aminoglycoside, NSAIDs treatment, ACE/ARB/ACEI treatment, contrast examination, Elixhauser comorbidity score, SOFA score, loop diuretic within 24 h of AKI, alert, hospital, and duration of AKI. The multivariate logistic regression analysis revealed that the complete group could decrease the 14-day AKI progression, 14-day dialysis, and 14-day mortality, with the adjusted OR values of 0.48 (95% CI: 0.33 to 0.70, *P* < 0.001), 0.26 (95% CI: 0.09 to 0.77, *P* = 0.015), and 0.53 (95% CI: 0.33 to 0.84, *P* = 0.006), respectively; and that the complete group could increase the discharge to home, the OR value was 1.50 (95% CI: 1.20 to 1.86, *P* < 0.001) (Table 2).

**Table 2:** Univariate and multivariate logistic regression analysis.

**Exposure**	**Unadjusted OR, (95% CI), *P* Value**	**Adjusted OR, (95% CI), *P* Value**
**14-day AKI progression**
Alert seen by attending physician	0.85 (0.68, 1.08), 0.183	1.34 (0.97, 1.85), 0.077
**Alert seen by attending physician**		
Partially	Reference	Reference
Completely	0.35 (0.25, 0.48), < 0.001	0.48 (0.33, 0.70), < 0.001
**14-day dialysis**
Alert seen by attending physician	0.99 (0.62, 1.58), 0.967	1.99 (0.96, 4.12), 0.065
**Alert seen by attending physician**		
Partially	Reference	Reference
Completely	0.16 (0.06, 0.44), < 0.001	0.26 (0.09, 0.77), 0.015
**14 day-mortality**
Alert seen by an attending physician	1.77 (1.33, 2.35), < 0.001	1.76 (1.11, 2.81), 0.017
**Alert seen by an attending physician**		
Partially	Reference	Reference
Completely	0.75 (0.55, 1.03), 0.078	0.53 (0.33, 0.84), 0.006
**Discharge to home**
Alert seen by attending physician	0.81 (0.68, 0.96), 0.013	1.42 (1.13, 1.80), 0.003
**Alert seen by attending physician**		
Partially	Reference	Reference
Completely	1.17 (0.99, 1.37), 0.063	1.50 (1.21, 1.87), < 0.001

Adjusted variables: Age, sex, race, Na+, K+, anion gap, HB, aminoglycoside, NSAIDs treatment, ACE/ARB/ACEI treatment, contrast examination, Elixhauser comorbidity score, SOFA score, any diuretic post 24 h after AKI, alert, hospital, and duration of alert.

### The results of subgroup analysis of multi-factor regression analysis

In this study, subgroup analysis was conducted based on whether the duration of the alert was more than 2 days and whether the patients received alert care or usual care. Alerts care was displayed each time the chart was opened, provided the patient continued to meet the criteria for AKI. Therefore, the longer duration of the alert might reflect the period of AKI. In comparison to usual care, the alert care featured an option to add AKI to the patient's issue list and a link to an AKI order set, which included choices for blood and urine testing as well as renal imaging but was restricted to minimal-risk tests and operations (that is, intravenous fluid administration was not included). When the rate of AKI alert seen by the attending physician as a continuous variable, it was found that the duration of the alert >2 days, the higher the alert seen by the attending physician, the higher the patient's 14-day mortality, with an OR value 2.67 (95% CI: 1.10 to 6.46, *P* = 0.029); While when the duration of alert ≤ 2 days, the higher the alert seen by attending physician, the higher the patient's discharge to home, with an OR value of 1.53 (95% CI: 1.18 to 1.99, *P* = 0.001); When the patients received usual care, the higher the alert seen by attending physician, the higher the patient's 14-day AKI progress and 14-day mortality, with OR values of 1.78 (95% CI: 1.05 to 3.00, *P* = 0.031) and 2.81 (95% CI: 1.46 to 5.44, *P* = 0.002), respectively. And when the patient received an alert, the higher the alert was seen by the attending physician, the higher the discharge to the home of the patient. When the rate of AKI alert seen by attending physical as a classified variable, it was found that when the duration of alert ≤ 2 days, the complete group significantly reduced 14-day AKI progress and 14-day mortality, with ORs 0.50 (95% CI: 0.28 to 0.89, *P* = 0.018) and 0.58 (95% CI: 0.34 to 0.99, *p* = 0.047), respectively. No matter the duration of alert ≤ 2 days or >2 days, the complete group significantly improved the discharge to home, with ORs of 1.54 (1.54 (95% CI: 1.21 to 1.95, *P* < 0.001) and 1.82 (1.82 (95% CI: 1.03 to 3.21, *P* = 0.038), respectively. When the patient received an alert, the complete group significantly reduced the 14-day mortality of the patient, with an OR value of 0.44 (95% CI: 0.22 to 0.87, *P* = 0.018). Regardless of whether the patient was with usual care or alert, the complete group significantly improved discharge to home, with ORs of 1.55 (1.55 (95% CI: 1.14 to 2.11, *P* = 0.005) and 1.59 (95% CI: 1.17 to 2.17, *P* = 0.003), respectively (Table 3).

**Table 3:** Subgroup analysis of multivariate logistic regression analysis.

**Exposure**	**Alert seen by attending physician^a^**	***P-*value**	**Alert seen by attending physician^b^**	***P*-value**
	**Adjusted OR(95% CI)**		**Adjusted OR(95% CI)**	
**14-day AKI progression**				
**Duration of alert**				
≤ 2 days	1.19 (0.71, 2.01)	0.504	0.50 (0.28, 0.89)	0.018
>2 days	1.47 (0.88, 2.45)	0.146	0.68 (0.38, 1.21)	0.186
**Alert**				
Usual care	1.78 (1.05, 3.00)	0.031	0.58 (0.32, 1.03)	0.062
Alert	1.05 (0.63, 1.75)	0.845	0.61 (0.35, 1.06)	0.082
**14-day dialysis**				
**Duration of alert**				
≤ 2 days	2.05 (0.49, 8.54)	0.324	0.63 (0.07, 5.38)	0.676
>2 days	2.53 (0.95, 6.77)	0.065	0.33 (0.08, 1.27)	0.106
**Alert**				
Usual care	2.72 (0.84, 8.78)	0.094	0.32 (0.06, 1.68)	0.180
Alert	2.07 (0.69, 6.23)	0.197	0.29 (0.06, 1.51)	0.142
**14-day mortality**				
**Duration of alert**				
≤ 2 days	1.61 (0.91, 2.84)	0.102	0.58 (0.34, 0.99)	0.047
>2 days	2.67 (1.10, 6.46)	0.029	0.54 (0.20, 1.45)	0.222
**Alert**				
Usual care	2.81 (1.46, 5.44)	0.002	0.69 (0.37, 1.28)	0.239
Alert	1.28 (0.65, 2.49)	0.472	0.44 (0.22, 0.87)	0.018
**Discharge to home**				
**Duration of alert**				
≤ 2 days	1.53 (1.18, 1.99)	0.001	1.54 (1.21, 1.95)	< 0.001
>2 days	1.25 (0.74, 2.09)	0.405	1.82 (1.03, 3.21)	0.038
**Alert**				
Usual care	1.25 (0.89, 1.74)	0.196	1.55 (1.14, 2.11)	0.005
Alert	1.65 (1.20, 2.28)	0.002	1.59 (1.17, 2.17)	0.003

a Alert seen by attending physician as a continuity variable.

b Alert seen by attending physician as a classification variable (the completely group vs. partially group, the partially group as a reference), the partial group was used as an control to calculate the risk ratios. Adjusted variables (without the subgroup analysis variables themselves): Age, sex, race, Na+, K+, anion gap, HB, aminoglycoside, NSAIDs treatment, ACE/ARB/ACEI treatment, contrast examination, Elixhauser comorbidity score, SOFA score, any diuretic post 24 h after AKI, alert, hospital, and duration of alert.

### The results of curve fitting and threshold effect analysis

**Figure 1:**
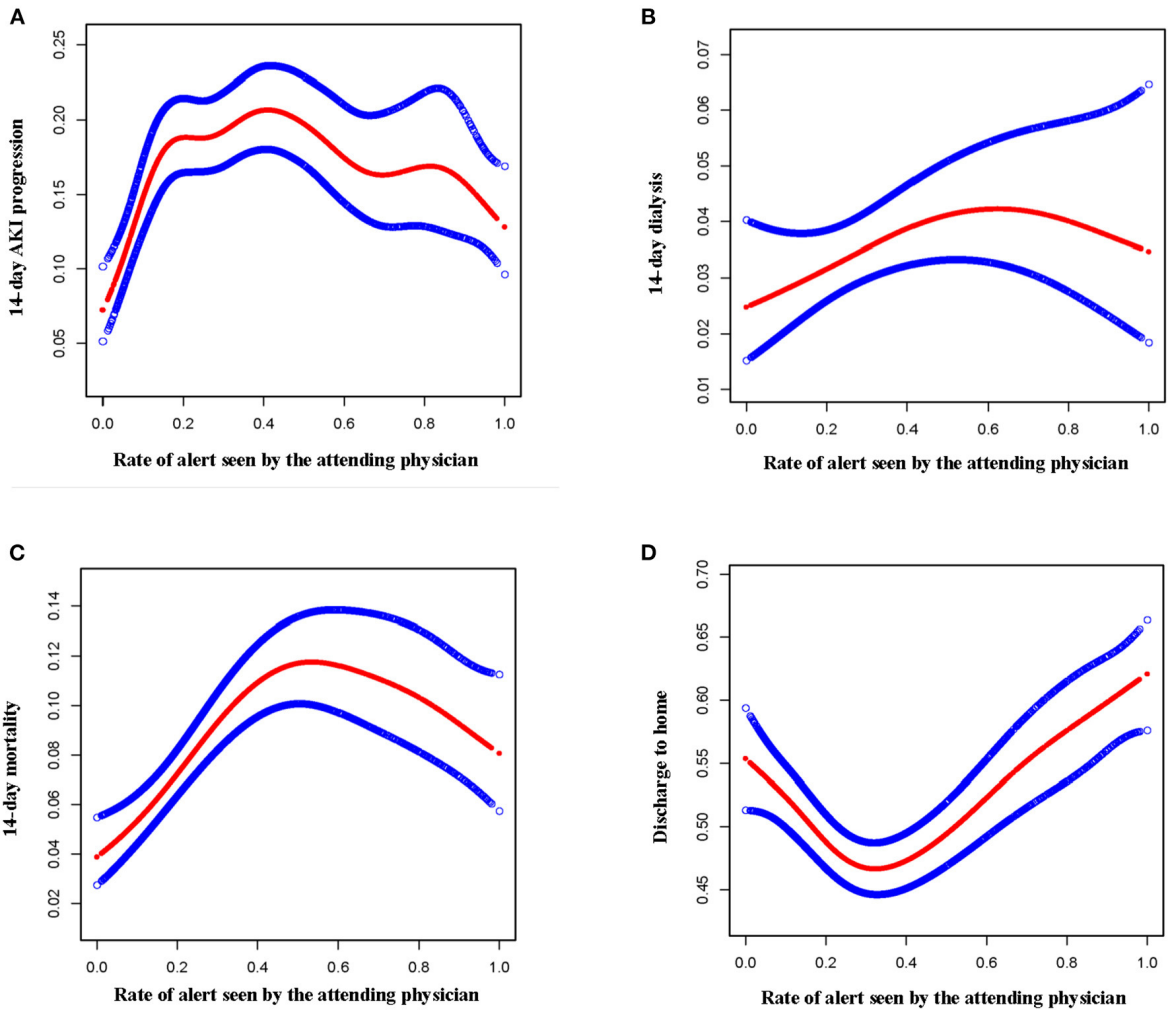
The results of curve fitting analysis. **(A)** The relationship between alert seen by attending physician and 14-day AKI progression. **(B)** The relationship between alert seen by attending physician and 14-day dialysis. **(C)** The relationship between alert seen by attending physician and 14-day mortality. **(D)** The relationship between alert seen by attending physician and the discharge to home.

Using curve fitting and threshold effect analysis, it was shown that when the alert seen by the attending physician < 10%, the 14-day AKI Progression increased dramatically. However, when the alert seen by an attending physician ≥10%, the 14-day AKI Progression did not rise significantly. 14-day dialysis rose when the alert was seen by the attending physician < 45%; conversely, 14-day dialysis reduced dramatically when seen by the attending physician ≥45%. When the alert was seen by the attending physician < 30%, the 14-day mortality rose considerably; however, the 14-day mortality did not increase when the alert was seen by the attending physician ≥30%. When an alert was seen by the attending physician < 29%, discharge to home dropped dramatically; While the proportion of alerts seen by the attending physician ≥29%, the proportion of patients discharged to their homes increased (Table 4 and Figure 1).

**Table 4:** The results of threshold effect analysis.

**Exposure**	**Unadjusted OR, (95% CI), *P*-value**	**Adjusted OR, (95% CI), *P*-value**
**14-day AKI progression**		
< 0%	Inf. (50426.53, inf.), < 0.001	Inf. (2908.74, inf.), < 0.001
≥10%	0.43 (0.32, 0.56), < 0.001	0.75 (0.51, 1.09), 0.131
**14-day dialysis**		
< 45%	56.38 (12.66, 251.04), < 0.001	43.38 (6.56, 286.89), < 0.001
≥45%	0.06 (0.02, 0.23), < 0.001	0.11 (0.02, 0.64), 0.014
**14-day mortality**		
< 30%	557.22 (85.60, 3627.33), < 0.001	183.23 (17.39, 1930.64) < 0.001
≥30%	0.69 (0.44, 1.09), 0.110	0.51 (0.24, 1.08), 0.078
**Discharge to home**		
< 29%	0.06 (0.03, 0.15), < 0.001	0.30 (0.11, 0.85), 0.023
≥29%	1.61 (1.23, 2.11), < 0.001	2.22 (1.51, 3.27), < 0.001

Alert seen by attending physician as a continuity variable in the threshold effect analysis.

## Discussion

This study discovered that the rate of alert seen by attending physicians is closely related to the prognosis of AKI patients. The higher the rate of alert seen by the attending physician, the lower the 14-day AKI progress, 14-day dialysis, and 14-day mortality of AKI patients, and the higher the discharge to home. Especially in the early stage of AKI, the higher the rate of alert seen by the attending physician, the better the prognosis of patients.

Currently, there were numerous research on AKI-related early warning models, but most of them had limited clinical importance due to their small sample size and lack of efficient external validation (15–17). The early warning model based on electronic medical data was capable of constantly validating and enhancing the model's prediction capacity as additional patients are added. Secondly, electronic medical records contained a wealth of clinical data, including demographic characteristics, disease characteristics, and related laboratory test results of patients, which made the alert model more reliable. More importantly, the electronic medical record-based prediction model was implanted into the electronic medical record system, allowing it to predict AKI automatically, and it was beneficial to guiding clinical practice (18). Unfortunately, two substantial studies on AKI early warning based on electronic medical records had shown that they cannot improve patients' prognoses (12, 13). We believed the probable explanations are as follows: The primary distinction between the alert group and the usual care group was that the alert group merely provided an AKI warning to on-duty physicians, but it did not mandate action based on an AKI warning, which might lead to the delay of AKI consultation and deterioration of patient prognosis (19). Unfortunately, this was not enough for the management of AKI. To effectively prevent or halt the course of AKI, a variety of systematic measures must be taken, such as modifying the dosage of a medication, avoiding addiction, and monitoring fluid balance, etc., (14). However, on-duty doctors were unable to complete a continuous and systematic AKI treatment, which might be the fundamental reason why electronic medical record alerts couldn't considerably improve the prognosis of AKI patients.

In the existing healthcare system, only the attending physician provided a systematic approach to AKI treatment. The only way to avoid the development of AKI and improve the prognosis of patients with AKI was to send AKI alerts continuously and effectively to attending doctors, who then constructed or changed the AKI treatment strategy dynamically based on the AKI alert (20).

In this study, we found that the higher the duration of alert ≤ 2 days, the higher the rate of alert seen by the attending physician, and the better the prognosis of patients. attending doctors could get an early warning of AKI and implement relevant therapies as soon as feasible, which might dramatically improve patients' prognoses. In the early phase of AKI (21), particularly within the first 48 h of AKI, by actively taking relevant treatment measures, such as maintaining hemodynamic stability, avoiding the continued use of nephrotoxic drug properties, and providing relevant renal protection, further deterioration of renal function and the worsening of patients' prognoses can be prevented.

In addition, this research identified a threshold relationship between the rate of alerts seen by the attending physician and the prognosis of patients. The thresholds for 14-day AKI progress, 14-day dialysis, 14-day mortality, and discharge to home are 10, 45, 30, and 29% respectively. Therefore, the rate of AKI alert by the attending physician should be improved as much as possible. It was unrealistic to let the attending doctor get all the alerts. The rate of AKI alert seen by the attending physician should be one of the characteristics included in the future early warning model, and our research may provide some reference for it.

Monique conducted a comprehensive analysis of articles related to clinical decision support systems and found that 57% of clinical decision support systems could affect the behavior of doctors, which in turn affected the prognosis of patients (22). At the same time, the rate of AKI alert seen by the attending physician would affect the treatment time of doctors after the occurrence of AKI to a certain extent. A prospective observational study by Kristen found that 28.7% of patients in the alert group received interventions, such as fluid therapy, diuretic, or vasopressors, and the interventions were significantly more effective than the control (17). Within 8 h= of the AKI notice, patients in the alert group had substantially higher rates of renal function recovery to baseline than patients in the control group (23). This showed that if the attending physician can receive and deal with the alert records in time, it will have a favorable impact on the prognosis of patients with AKI. Therefore, improving the awareness rate of AKI alerts by attending physicians was the primary key to ensuring the prognosis of patients with AKI.

### Application value of the research

Electronic medical records contained a wealth of clinical data. This study discovered that the electronic medical record-based prediction model was implanted into the electronic medical record system, allowing it to predict AKI automatically, and it was beneficial to guiding clinical practice. The rate of alert seen by the attending physicians was closely related to the prognosis of AKI patients. Attending doctors could construct or change the AKI treatment strategy dynamically based on the AKI alert. Especially in the early stage of AKI, they could get an early warning of AKI and take actively relevant treatment measures to provide relevant renal protection. In addition, this research identified a threshold relationship between the rate of alerts seen by the attending physician and the prognosis of patients. These results could provide some references for future research on related early warning models.

### Limitations of the study

This research belongs to the second retrospective analysis of data; hence the result of this study should be validated by subsequent prospective investigations. Due to the fact that creatinine was used as the only indicator of AKI definition in this electronic early warning system and urine volume was ignored, the population of people with AKI in this study might be underestimated, and the research could not rule out the impact of different clinical departments, such as critical care or surgery, as well as the type of AKI (prerenal, renal, or posterior) on the progression or prognosis of AKI patients, introducing the possibility of bias into the findings of this study. The influence of the rate of AKI alert detection by attending physicians on the alteration of medical behavior and the choice of treatment method needs to be further validated, and the causal link between them is required to be further proven. The role of a clinical nephrologist in AKI alert was not investigated in this research. If an AKI alert was sent to a clinical nephrologist, the patient's clinical prognosis might be improved.

## Conclusion

In conclusion, the study revealed a correlation between the rate of AKI alert detection by the attending physician and the prognosis of the patient. The prognosis of patients with AKI improves with a greater probability of AKI alarm identification by the attending physician. The better the prognosis of patients, particularly in the early stages of AKI, the greater the rate of alertness seen by the attending physician. They may get an early warning of AKI and adopt actively appropriate therapeutic steps to successfully prevent or arrest the progression of AKI, particularly in the early stages of AKI. In clinical practice, we must thus increase the proportion of AKI alert detection by the attending physician. Whether it is the nursing team or the doctor team, if an AKI alert is detected, they should immediately notify the attending physician so that the attending physician can grasp the progression of the patient's condition, which makes the electronic alarm system a vital tool for assisting doctors with diagnosis and treatment.

## Data availability statement

The datasets presented in this study can be found in online repositories. The names of the repository/repositories and accession number(s) can be found below: https://datadryad.org/stash/dataset/doi:10.5061/dryad.4f4qrfj95.

## Ethics statement

Ethical review and approval was not required for the study on human participants in accordance with the local legislation and institutional requirements. Written informed consent for participation was not required for this study in accordance with the national legislation and the institutional requirements.

## Author contributions

YS and HW participated in the research design, the writing of the manuscript, and data analysis. LB, YW, and LZ participated in data analysis. XZ and J-hL participated in the improving and revising of the paper. H-hP and Z-hB provided substantial advice in designing the study and assisting in the division of labor, writing, and revising the paper. All authors contributed to the article and approved the submitted version.

## Funding

Key R&D projects of Shaanxi Province, funding number: 2022SF-568.

## Conflict of interest

The authors declare that the research was conducted in the absence of any commercial or financial relationships that could be construed as a potential conflict of interest.

## Publisher's note

All claims expressed in this article are solely those of the authors and do not necessarily represent those of their affiliated organizations, or those of the publisher, the editors and the reviewers. Any product that may be evaluated in this article, or claim that may be made by its manufacturer, is not guaranteed or endorsed by the publisher.

## Abbreviations

NSAIDs: non-steroidal anti-inflammatory drugs
MAP: mean arterial pressure
CKD: chronic kidney disease
COPD: chronic obstructive pulmonary disease
BUN: Blood urea nitrogen
SOFA: sequential organ failure assessment
AKI: acute kidney injury
ACEi: angiotensin-converting enzyme inhibitors
ARB: angiotensin receptor blocker
KDIGO: Kidney Disease: Improving Global Outcomes.
